# 

**Published:** 1916-06

**Authors:** 


**CONTENTS, JUNE, 1916—VOLUME XXXI, NO. 12.**

ORIGINAL ARTICLES.

The Diagnosis of Internal Secretory Disorders. (Article No. 2.) By Dr. Henry R. Harrower, M. D., F. R. S. N., Los Angeles, California ........ 487

Vaccines in Mixed Pulmonary Infection. By Theo. Y. Hull, M. D., San Antonio, Texas ........ 491

Congenital Pyloric Stenosis. By Joe Gilbert, M. D., Austin, Texas ........ 499

Some Observations on Childhood. By E. S. Goodhue, A. M., M. D., LL. D. ........ 503

Twilight Sleep. By Dr. J. B. Dorrell, Springfield, Mo ........ 509

EDITORIAL DEPARTMENT.

The Physician's Opportunity ........ 513

Some Things for the Future ........ 513

Pellagra ........ 515

Items of General Interest ........ 518

Personal Mention ........ 524

Deaths ........ 526

Helpful Hints for the Busy Doctor ........ 526

